# A Berry-Esseen type bound for the kernel density estimator based on a weakly dependent and randomly left truncated data

**DOI:** 10.1186/s13660-016-1272-0

**Published:** 2017-01-03

**Authors:** Petros Asghari, Vahid Fakoor

**Affiliations:** Department of Statistics, Faculty of Mathematical Sciences, Ferdowsi University of Mashhad, P.O. Box 1159-91775, Mashhad, Iran

**Keywords:** left-truncation, weakly dependent, asymptotic normality, Berry-Esseen, *α*-mixing

## Abstract

In many applications, the available data come from a sampling scheme that causes loss of information in terms of left truncation. In some cases, in addition to left truncation, the data are weakly dependent. In this paper we are interested in deriving the asymptotic normality as well as a Berry-Esseen type bound for the kernel density estimator of left truncated and weakly dependent data.

## Introduction

$\mathcal{P}$ is a population with large, deterministic and finite size *N* with elements $\{ ( Y_{i}, T_{i}); i=1,\ldots,N\}$. In sampling from this population we only observe those pairs for which $Y_{i} \geq T_{i}$. Suppose that there is at least one pair with this condition. The sample is denoted by $\{ (Y_{i}, T_{i}); i=1,\ldots,n\}$. This model is called random left-truncated model (RLTM). We assume that $\{ Y_{i}; i \geq1 \}$ is a stationary *α*-mixing sequence of random variables and $\{ T_{i}; i=1, \ldots,N\}$ is an independent and identically distributed (i.i.d.) sequence of random variables. The definition of a strong mixing sequence is presented in Definition 1.

### Definition 1

Let ${ \{ {{Y_{i}; i \ge1}} \}}$ be a sequence of random variables. The mixing coefficient of this sequence is

$$\alpha ( m ) = \sup_{k \ge1} \bigl\{ {\bigl\vert {\mathrm{P} ( {A \cap B} ) - \mathrm{P} ( A )\mathrm{P} ( B )} \bigr\vert ; A \in{ \mathcal{F}}_{1}^{k}, B \in{\mathcal{F}}_{k + m}^{\infty}} \bigr\} , $$

where ${\mathcal{F}}_{l}^{m}$ denotes the *σ*-algebra generated by $\{ {{Y_{j}}} \}$ for $l \le j \le m$. This sequence is said to be strong mixing or *α*-mixing if the mixing coefficient converges to zero as $m\rightarrow\infty$.

Studying the various aspects of left-truncated data is of high interest due to their applicability in much research. One of these applications is in survival analysis. It is well known that in medical research on some specific diseases such as AIDS and dementia, the sampling scheme results in data samples that are left truncated. This model also arises in astronomy [1].

Strong mixing sequences of random variables are widely occurring in practice. One application is in the analysis of time series and in renewal theory. A stationary ARMA-sequence fulfils the strong mixing condition with an exponential rate of mixing coefficient. The concept of strong mixing sequences was first introduced by Rosenblatt [2] where a central limit theorem is presented for a sequence of random variables that satisfies the mixing condition.

The Berry-Esseen inequality or theorem was stated independently by Berry [3] and Esseen [4]. This theorem specifies the rate at which the scaled mean of a random sample converges to the normal distribution for all sample spaces. Parzen [5] derived a Berry-Esseen inequality for the kernel density estimator of an i.i.d. sequence of random variables. Several works were done for left-truncated observations. We can refer to [6] where the distribution of left-truncated data was estimated and asymptotic properties of the estimator were derived. More work was done by Stute [7]. Prakasa Rao [8] presented a Berry-Esseen theorem for the density estimator of a sample that forms a stationary Markov process. Liang and Un̈a-Álvarez [9] have derived a Berry-Esseen inequality for mixing data that are right censored. Yang and Hu [10] presented Berry-Esseen type bounds for kernel density estimator based on a *φ*-mixing sequence of random variables. Asghari *et al.* [11, 12] presented a Berry-Esseen type inequality for the kernel density estimator, respectively, for a left-truncated model and for length-biased data.

This paper is organized as follows. In Section 2, needed notations are introduced and some preliminaries are listed. In Section 3, the Berry-Esseen type theorem for the estimator of the density function of the data is presented. In Section 4, the theorems and corollaries of Section 3 are proved.

## Preliminaries and notation

Suppose that $Y_{i}$’s and $T_{i}$’s for $i=1, \ldots,N$ are positive random variables with distributions *F* and *G*, respectively. Let the joint distribution function of $( Y_{1}, T_{1} )$ be

$$\begin{aligned} {H^{*}} ( {y,t} ) = &\mathrm{P} ( Y_{1} \le y, T_{1} \le t ) \\ = &\frac{1}{\alpha} \int_{ - \infty}^{y} {G ( {t \wedge u} )\,dF ( u )}, \end{aligned}$$

in which $\alpha=\mathrm{P} ( Y_{1}\geq T_{1} )$.

If the marginal distribution function of $Y_{i}$ is denoted by $F^{*}$, we have

$${F^{*}} ( y ) = \frac{1}{\alpha} \int_{ - \infty}^{y} {G ( u )\,dF ( u )}, $$

so the marginal density function of *Y* is

$${f^{*}} ( y ) = \frac{1}{\alpha}G ( y )f ( y ). $$

A kernel estimator for *f* is given by

$${f_{n}} ( y ) = \frac{1}{{n{h_{n}}}}\sum_{i = 1}^{n} {K \biggl( {\frac{{{Y_{i}} - y}}{{{h_{n}}}}} \biggr)\frac{\alpha}{{G ( {{Y_{i}}} )}}}. $$

In many applications, the distribution function of the truncation random variable *G* is unknown. So $f_{n} ( y )$ is not applicable in these cases and we need to use an estimator of *G*. Before starting the estimation details, for any distribution function *L* on $[0,\infty]$, let $a_{L}:=\inf\{x>0: L(x)>0\}$ and $b_{L}:=\sup\{x>0: L(x)<1\}$.

Woodroof [6] pointed out that *F* and *G* can be estimated only if $a_{G} \leq a_{F}$, $b_{G} \leq b_{F}$ and $\int_{{a_{F}}}^{\infty}{\frac {{dF}}{G}} < \infty$. This integrability condition can be replaced by the stronger condition $a_{G} < a_{F}$. Using this assumption, here we use the non-parametric maximum likelihood estimator for *G* that is presented by Lynden-Bell [13] and is denoted by $G_{n}$,

2.1 $$ {G_{n}} ( y ) = \prod_{i: Y_{i} > y} { \biggl( {1 - \frac {{S(y)}}{{{C_{n}}(y)}}} \biggr)}, \quad 0\leq y < \infty, $$

in which ${S}(y) = \sum_{i = 1}^{n} {I_{ \{ Y_{i} = y \}}} $ and $C_{n}(s) = \frac{1}{n}\sum_{i = 1}^{n} {{I_{ \{ {{T_{i}} \le s \le{Y_{i}}} \}}}} $.

Using the definition of $C_{n}$ that is mentioned in the estimation procedure of *G* and also using the empirical estimators of $F^{*}$ and $G^{*}$, which are denoted by $F_{n}^{*}$ and $G_{n}^{*}$, we have

$$C_{n}(y) = G_{n}^{*} ( y ) - F_{n}^{*} ( y ) ,\quad y \in [{a_{F}}, + \infty). $$

It can be seen that ${C_{n}} ( s )$ is actually the empirical estimator of $C_{n} = {G^{*}} ( y ) - {F^{*}} ( y ) = {\alpha^{ - 1}}G ( y ) [ {1 - F ( y )} ]$, $y \in[{a_{F}}, + \infty)$. This fact gives the following estimator of *α*:

$${\alpha_{n}} = \frac{{{G_{n}} ( y ) [ {1 - {F_{n}} ( y )} ]}}{C_{n}(y)}. $$

For details as regards $\alpha_{n} $, see [14]. Using $\alpha_{n} $, we present a more applicable estimator of *f*, which is denoted $\hat{f}_{n}$ and is defined as

2.2 $$ {{\hat{f}}_{n}} ( y ) = \frac{1}{{n{h_{n}}}}\sum _{i = 1}^{n} {K \biggl( {\frac{{{Y_{i}} - y}}{{{h_{n}}}}} \biggr) \frac{{{\alpha _{n}}}}{{{G_{n}} ( Y_{i} )}}}. $$

Note that in (2.2) the sum is taken over *i*’s for which ${G_{n}} ( {{Y_{i}}} ) \ne0$.

## Results

Before presenting the main theorems, we need to state some assumptions. Suppose that $a_{G} < a_{F}$ and $b_{G} \leq b_{F}$. Woodroof [6] stated that the uniform convergence rate of $G_{n}$ to *G* is true for $y \in [ {a,{b_{G}}} ]$ for $a>a_{G}$. Thus, we have to assume that $a_{G} < a_{F}$. Let $\mathcal{C}= [ {a,b} ]$ be a compact set such that $\mathcal{C} \subset \{ y;y \in [ {{a_{F}},{b_{F}}} [ \}$. As mentioned in the Introduction, $\{ Y_{i}; i \geq1 \}$ is a stationary *α*-mixing sequence of random variables with mixing coefficient $\beta(n)$, and $\{ T_{i}; i \geq1\}$ is an i.i.d. sequence of random variables.

### Definition 2

The kernel function *K*, is a second order kernel function if $\int _{-\infty}^{\infty}{K ( t )\,dt} = 1$, $\int_{-\infty}^{\infty} {tK ( t )\,dt} = 0$ and $\int_{-\infty}^{\infty} {{t^{2}}K ( t )\,dt} > 0$.

### Assumptions

A1: $\beta(n)=O ( n^{-\lambda} )$ for some $\lambda> \frac{2+\delta}{\delta}$ in which $0 < \delta\leq1$.
A2: For the conditional density of $Y_{j+1}$ given $Y_{1}=y_{1}$ (denoted by $f_{j} ( \cdot|y_{1} )$), we have $f_{j} ( y_{2} | y_{1} ) \leq M $ for $y_{1}$ and $y_{2}$ in a neighborhood of $y\in\mathbb {R}$ in which *M* is a positive constant.
A3
  (i) *K* is a positive bounded kernel function such that $K ( t )=0$ for $\vert t\vert >1$ and $\int_{ - 1}^{1} {K ( t )} = 1$.
  (ii) *K* is a second order kernel function.
  (iii) *f* is twice continuously differentiable.
A4: Let $p=p_{n}$ and $q=q_{n}$ be positive integers such that $p+q \leq n$, there exists a constant *C* such that for *n* large enough $\frac{q}{p} \le C$. Also $p{h_{n}} \to0$, $q{h_{n}} \to0$ as $n \to \infty$.
A5: $\{ {{T_{i}};i \ge1} \}$ is a sequence of i.i.d. random variable with common continuous distribution function *G*, and independent of $\{ {{Y_{i}};i \ge1} \}$.
H1: The kernel function $K( \cdot)$ is differentiable and Hölder continuous with exponent $\beta> 0$.
H2: $\beta ( n )=O ( n^{-\lambda} )$ for $\lambda > \frac{{1 + 5\beta}}{\beta}$ in which $\beta> \frac{1}{7}$.
H3: The joint density of $( Y_{i} , Y_{j} )$, $f_{ij}^{*}$, exists and we have $\sup_{u,v} \vert {f_{ij}^{*} ( {u,v} ) - {f^{*}} ( u ){f^{*}} ( v )} \vert \le C < \infty$ for some constant *C*.
H4: There exists $\lambda>5+\frac{1}{\beta}$ and for the bandwidth $h_{n}$ we have $\frac{\log\log n}{nh_{n}^{2}} \to0$ and $C{n^{\frac{{ ( {3 - \lambda} )\beta}}{{\beta ( {\lambda + 1} ) + 2\beta + 1}} + \eta}} < {h_{n}} < C'{n^{\frac{1}{{1 - \lambda}}}}$ which *η* is such that $\frac{1}{{\beta ( {\lambda + 1} ) + 2\beta + 1}} < \eta < \frac{{ ( {\lambda - 3} )\beta}}{{\beta ( {\lambda + 1} ) + 2\beta + 1}} + \frac{1}{{1 - \lambda}}$.

*Discussion of the assumptions*. A1, A2, and A4 are common in the literature. For example Zhou and Liang [15] used A2 for deconvolution estimator of multivariate density of *α*-mixing process. A3(i)-(ii) are commonly used in non-parametric estimation. A3(iii) is specially needed for a Taylor expansion. H1-H4 are needed to use Theorem 4.1 of [16] in Theorem 4 here.

Let $\sigma_{n}^{2} ( y ) := n{h_{n}}\mathit {Var}[ {{f_{n}} ( y )} ]$, so by letting $\frac{1}{{\sqrt{n{h_{n}}} }}K ( {\frac{{{Y_{i}} - y}}{{{h_{n}}}}} )\frac{\alpha}{{G ( {{Y_{i}}} )}} =: {W_{ni}}$, we can write

3.1 $$\begin{aligned} \sigma_{n}^{2} ( y ) =& \mathit {Var}\Biggl( {\sum _{i = 1}^{n} {\frac {1}{{\sqrt{n{h_{n}}} }}K \biggl( { \frac{{{Y_{i}} - y}}{{{h_{n}}}}} \biggr)\frac {\alpha}{{G ( {{Y_{i}}} )}}} } \Biggr) \\ =& \mathit {Var}\Biggl( {\sum_{i = 1}^{n} {{W_{ni}}} } \Biggr). \end{aligned}$$

Let $k = [ {\frac{n}{{p + q}}} ]$, ${k_{m}} = ( {m - 1} ) ( {p + q} ) + 1$ and ${l_{m}} = ( {m - 1} ) ( {p + q} ) + p + 1$, in which $m=1,2,\ldots,k$. Now we have the following decomposition:

3.2 $$ \sum_{i = 1}^{n} {{W_{ni}}}=\mathcal{J}'_{n}+\mathcal {J}''_{n}+\mathcal{J}'''_{n}, $$

in which

$$\begin{aligned}& \mathcal{J}'_{n}=\sum_{m = 1}^{k} {{j'_{nm}}}, \quad\quad {j'_{nm}} = \sum _{i = {k_{m}}}^{{k_{m}} + p - 1} {{W_{ni}}}, \\& \mathcal{J}''_{n}=\sum _{m = 1}^{k} {{j''_{nm}}},\quad\quad {j''_{nm}} = \sum _{i = {l_{m}}}^{{l_{m}} + q - 1} {{W_{ni}}}, \\& \mathcal{J}'''_{n}={j''_{nk + 1}},\quad\quad {j''_{nk + 1}} = \sum _{i = k ( {p + q} ) + 1}^{n} {{W_{ni}}}. \end{aligned}$$

From now on, we let ${\sigma^{2}}(y):=\frac{\alpha f (y)}{G(y)}\int_{-1}^{1} {{K^{2}}(t)\,dt} $, $u(n):=\sum_{j= n}^{\infty}{(\alpha (j))^{\frac{\delta}{\delta + 2}}}$.

### Theorem 1

*If Assumptions* A1-A3(i) *and* A4 *are satisfied and* *f* *and* *G* *are continuous in a neighborhood of* *y* *for* $y \geq a_{F}$, *then for large enough* *n* *we have*

$$\sup_{x \in\mathbb{R}} \bigl\vert {\mathrm{P} \bigl( {\sqrt {n{h_{n}}} \bigl[ {{f_{n}} ( y ) - E{f_{n}} ( y )} \bigr]} \le x{ \sigma_{n}} ( y ) \bigr)- \Phi ( x )} \bigr\vert = O ( {a_{n}} ) \quad \textit{a.s.} $$

*in which*

3.3 $$ {a_{n}} = h_{n}^{\frac{{2 ( {1 + \delta} )}}{{2 + \delta }}}{ \biggl( { \frac{p}{{n{h_{n}}}}} \biggr)^{1 + \delta'}} + { \bigl( {{\lambda'''_{n}} \alpha ( q )} \bigr)^{1/4}} + \lambda_{n}^{\prime\prime 1/2} + \lambda_{n}^{\prime\prime\prime1/2} + h_{n}^{ - \delta / ( 2 + \delta)} u ( q ), $$

*and*

3.4 $$ \begin{aligned} {\lambda''_{n}} &:= \frac{{kq}}{n} + h_{n}^{ - \delta/ ( 2 + \delta)} u ( q ) + q{h_{n}} , \\ {\lambda'''_{n}} &:= \frac{p}{n} ( {p{h_{n}} + 1} ) . \end{aligned} $$

### Theorem 2

*If the assumptions of Theorem* 1 *and* A5 *are satisfied*, *then for* $y \geq a_{F}$ *and for large enough* *n* *we have*

$$\sup_{x \in\mathbb{R}} \bigl\vert \mathrm{P} \bigl( \sqrt{ nh_{n} } \bigl[ {\hat{f}}_{n}( y ) - E\bigl( f_{n}( y ) \bigr) \bigr] \leq x {\sigma_{n}}( y ) \bigr) - \Phi ( x ) \bigr\vert = O \bigl( a_{n} + ( h_{n}\log\log n )^{ 1/4} \bigr) \quad \textit{a.s.} $$

*in which* $a_{n}$ *is defined in* (3.3).

### Theorem 3

*If the assumptions of Theorem* 2 *are satisfied*, *G* *has bounded first derivative in a neighborhood of* *y* *and* *f* *has bounded derivative of order *2 *in a neighborhood of* *y* *for* $y \geq a_{F}$, *then for large enough* *n* *we have*

$$\sup_{x \in\mathbb{R}} \bigl\vert {\mathrm{P} \bigl( { \sqrt{n{h_{n}}} \bigl[ {{{\hat{f}}_{n}} ( y ) - f ( y )} \bigr] \le x\sigma ( y )} \bigr) - \Phi ( x )} \bigr\vert = O \bigl( a'_{n} \bigr) \quad \textit{a.s.}, $$

*in which*

3.5 $$\begin{aligned} a'_{n} :=& h_{n}^{\frac{{2 ( {1 + \delta} )}}{{2 + \delta }}}{ \biggl( {\frac{p}{{n{h_{n}}}}} \biggr)^{1 + \delta'}} + {h_{n}} ( {p + 1} ) + h_{n}^{\frac{ - \delta}{2 + \delta}}u ( q ) + \bigl( {{\lambda'''_{n}} \alpha ( q )} \bigr)^{1/4} \\ & {}+ \lambda_{n}^{\prime\prime 1/2}+ \lambda_{n}^{\prime\prime\prime1/2} + \lambda _{n}^{\prime\prime 1/2}\lambda_{n}^{\prime\prime\prime1/2}, \end{aligned}$$

*and* $\lambda''$ *and* $\lambda'''$ *are defined in* (3.4).

### Remark 1

In many applications, *f* and *G* are unknown and should be estimated, so ${\sigma^{2}} ( y )$ is not applicable in these cases. Here we present an estimator for it that is denoted by $\hat{\sigma}_{n}^{2} ( y )$ and is defined as follows:

$$\hat{\sigma}_{n}^{2} ( y ) = \frac{{{\alpha_{n}}{{\hat{f}}_{n}} ( y )}}{{{G_{n}} ( y )}} \int_{ - 1}^{1} {{K^{2}} ( t )\,dt}. $$

Using this estimator instead of ${\sigma^{2}} ( y )$ in Theorem 3, costs a change in the rate of convergence. This change is discussed in the following corollaries.

### Corollary 1

*Let Assumptions* A3, A5 *and* H1-H4 *be satisfied*, *then for* $y \in\mathcal{C}$

$$\sup_{y \geq a_{F}} \bigl\vert {\hat{\sigma}_{n}^{2} ( y ) - {\sigma^{2}} ( y )} \bigr\vert = O ( c_{n} ) \quad \textit{a.s.}, $$

*in which*

3.6 $$ c_{n} := {\max \biggl( {\sqrt{\frac{{\log n}}{{n{h_{n}}}}} ,h_{n}^{2}} \biggr) + \sqrt{\frac{{\log\log n}}{n}} }. $$

### Theorem 4

*Let Assumptions* A1-A5 *and* H1-H4 *be satisfied*. *For* $y \in\mathcal{C}$ *and for large enough* *n* *we have*

$$\sup_{x \in\mathbb{R}} \bigl\vert \mathrm{P} \bigl( {\sqrt{nh_{n}} \bigl( {{{\hat{f}}_{n}}(y) - f(y)} \bigr)} \le x {{\hat{\sigma}}_{n}} ( y ) \bigr) - \Phi ( x ) \bigr\vert = O \bigl( a'_{n} + c_{n} \bigr) \quad \textit{a.s.}, $$

*in which* $a'_{n}$ *is defined in* (3.5) *and* $c_{n}$ *is defined in* (3.6).

## Proofs

In order to start the proofs of the main theorems, we shall state some lemmas that are used in the proving procedure of the main theorems. For the sake of simplicity let *C*, $C'$ and $C''$, be positive appropriate constants which may take different values at different places.

### Lemma 1

[17]

*Let* *X* *and* *Y* *be random variables such that* $E{\vert X \vert ^{r}} < \infty$ *and* $E{\vert Y \vert ^{s}} < \infty$ *in which* *r* *and* *s* *are constants such that* $r,s>1$ *and* ${r^{ - 1}} + {s^{ - 1}} < 1$. *Then we have*

$$\bigl\vert {E ( {XY} ) - E ( X )E ( Y )} \bigr\vert \le{ \Vert X \Vert _{r}} {\Vert Y \Vert _{s}} { \Bigl[ {\sup _{A \in\sigma ( X ),B \in\sigma ( Y )} \bigl\vert {\mathrm{P} ( {A \cap B} ) - \mathrm{P} ( A )\mathrm{P} ( B )} \bigr\vert } \Bigr]^{1 - 1 / r - 1 / s} }. $$

### Lemma 2

*Suppose that Assumptions* A1-A3(i) *and* A4 *are satisfied*. *If* *f* *and* *G* *are continuous in a neighborhood of* *y* *for* $y \geq a_{F}$ *then* $\sigma_{n}^{2} ( y ) \to{\sigma^{2}} ( y ) $ *as* $n \to\infty$. *Furthermore*, *if* *f* *and* *G* *have bounded first derivatives in a neighborhood of* *y* *for* $y\geq a_{F}$, *for such* *y’s we have*

$$\bigl\vert {\sigma_{n}^{2} ( y ) - {\sigma^{2}} ( y )} \bigr\vert = O ( {b_{n}} ), $$

*in which*

$${b_{n}} := {h_{n}} ( { p + 1} ) + h_{n}^{ - \delta/ ( 2 + \delta)} u ( q ) + \lambda_{n}^{\prime\prime1/2} + \lambda_{n}^{\prime\prime\prime1/2} + \lambda_{n}^{\prime\prime1/2} \lambda_{n}^{\prime\prime\prime1/2} , $$

### Proof

Using the decomposition that is defined in (3.2) we can write

4.1 $$\begin{aligned}& \sigma_{n}^{2} ( y ) = \mathit {Var}\bigl( {{{\mathcal {J}}' _{n}} + {{\mathcal {J}}'' _{n}} + {{\mathcal {J}}''' _{n}}} \bigr) \\& \hphantom{ \sigma_{n}^{2} ( y )}= \mathit {Var}\bigl( {{{\mathcal {J}}' _{n}}} \bigr) + \mathit {Var}\bigl( {{{\mathcal {J}}'' _{n}}} \bigr) + \mathit {Var}\bigl( {{{\mathcal {J}}''' _{n}}} \bigr) \\& \hphantom{ \sigma_{n}^{2} ( y )}\quad {} + 2\mathit {Cov}\bigl( {{{\mathcal {J}}' _{n}},{{\mathcal {J}}'' _{n}}} \bigr) + 2\mathit {Cov}\bigl( {{{\mathcal {J}}' _{n}},{{\mathcal {J}}''' _{n}}} \bigr) + 2\mathit {Cov}\bigl( {{{\mathcal {J}}'' _{n}},{{\mathcal {J}}''' _{n}}} \bigr), \end{aligned}$$

4.2 $$\begin{aligned}& \mathit {Var}\bigl( {{{\mathcal {J}}' _{n}}} \bigr) = \mathit {Var}\Biggl( {\sum_{m = 1}^{k} {{j'_{nm}}} } \Biggr) \\& \hphantom{ \mathit {Var}\bigl( {{{\mathcal {J}}'_{n}}} \bigr)}= \sum_{m = 1}^{k} {\sum _{i = {k_{m}}}^{{k_{m}} + p - 1} {\mathit {Var}( {{W_{ni}}} )} } \\& \hphantom{ \mathit {Var}\bigl( {{{\mathcal {J}}'_{n}}} \bigr)}\quad{} + 2\sum_{1 \le i < j \le k} {\mathit {Cov}\bigl( {{j'_{ni}},{j'_{nj}}} \bigr) + 2\sum_{m = 1}^{k} {\sum _{{k_{m}} \le i < j \le {k_{m}} + p - 1} {\mathit {Cov}( {{W_{ni}},{W_{nj}}} )} } } \\& \hphantom{ \mathit {Var}\bigl( {{{\mathcal {J}}'_{n}}} \bigr)}=: \mathrm{I}' + \mathrm{II}' + \mathrm{III}'. \end{aligned}$$

As assumed in the lemma, *f* and *G* are continuous in a neighborhood of *y* so they are bounded in this neighborhood. Now under Assumption A3(i) we have

4.3 $$\begin{aligned} \mathit {Var}( {{W_{ni}}} ) =& \frac{1}{{n{h_{n}}}} \biggl\{ {E \biggl[ {{K^{2}} \biggl( {\frac{{{Y_{i}} - y}}{{{h_{n}}}}} \biggr)\frac{{{\alpha ^{2}}}}{{{G^{2}} ( {{Y_{i}}} )}}} \biggr] - {E^{2}} \biggl[ {K \biggl( {\frac{{{Y_{i}} - y}}{{{h_{n}}}}} \biggr) \frac{\alpha}{{G ( {{Y_{i}}} )}}} \biggr]} \biggr\} \\ =& \frac{1}{{n{h_{n}}}} \biggl\{ { \int{{K^{2}} \biggl( {\frac{{u - y}}{{{h_{n}}}}} \biggr) \frac{{\alpha f ( u )}}{{G ( u )}}\,du - {{ \biggl[ { \int{K \biggl( {\frac{{u - y}}{{{h_{n}}}}} \biggr)f ( u )\,du} } \biggr]}^{2}}} } \biggr\} \\ =& \frac{1}{n} \biggl\{ { \int{{K^{2}} ( t )\frac{{\alpha f ( {y + t{h_{n}}} )}}{{G ( {y + t{h_{n}}} )}}\,dt - {h_{n}} {{ \biggl[ { \int{K ( t )f ( {y + t{h_{n}}} )\,dt} } \biggr]}^{2}}} } \biggr\} , \end{aligned}$$

so it can be concluded that

4.4 $$\begin{aligned} \bigl\vert {\mathrm{I}'} \bigr\vert \le& \frac{{kp}}{n} \biggl\{ { \int_{ - 1}^{1} {{K^{2}} ( t ) \frac{{\alpha f ( {y + t{h_{n}}} )}}{{G ( {y + t{h_{n}}} )}}\,dt} + {h_{n}} {{ \biggl[ { \int_{ - 1}^{1} {K ( t )f ( {y + t{h_{n}}} ) \,dt} } \biggr]}^{2}}} \biggr\} \\ =& O \biggl( {\frac{{kp}}{n}} \biggr). \end{aligned}$$

Lemma 1 for arbitrarily $\delta> 0$ and also the continuity of *f* in a neighborhood of *y* gives

$$\begin{aligned} \bigl\vert \mathrm{II}' \bigr\vert \leq& 2\sum _{1 \le i < j \le k} {\sum_{s = {k_{i}}}^{{k_{i}} + p - 1} {\sum_{t = {k_{j}}}^{{k_{j}} + p - 1} {\bigl\vert {\mathit {Cov}( {{W_{ns}},{W_{nt}}} )} \bigr\vert } } } \\ \le& \frac{C}{{n{h_{n}}}}\sum_{1 \le i < j \le k} {\sum _{s = {k_{i}}}^{{k_{i}} + p - 1} {\sum_{t = {k_{j}}}^{{k_{j}} + p - 1} {{{\biggl\Vert {K \biggl( {\frac{{{Y_{s}} - y}}{{{h_{n}}}}} \biggr)\frac{\alpha }{{G ( {{Y_{s}}} )}}} \biggr\Vert }_{2 + \delta}} {{\biggl\Vert {K \biggl( {\frac{{{Y_{t}} - y}}{{{h_{n}}}}} \biggr)\frac{\alpha}{{G ( {{Y_{t}}} )}}} \biggr\Vert }_{2 + \delta}}} } } \\ & {}\times{ \bigl( {\alpha ( {t - s} )} \bigr)^{1 - 2/ ( 2 + \delta)} } \\ =& \frac{C}{{n{h_{n}}}}\sum_{1 \le i < j \le k} {\sum _{s = {k_{i}}}^{{k_{i}} + p - 1} {\sum_{t = {k_{j}}}^{{k_{j}} + p - 1} {\biggl\Vert {K \biggl( {\frac{{{Y_{1}} - y}}{{{h_{n}}}}} \biggr)\frac{\alpha}{{G ( {{Y_{1}}} )}}} \biggr\Vert _{2 + \delta}^{2}{ \bigl( {\alpha ( {t - s} )} \bigr)^{\delta / ( 2 + \delta )} }} } } \\ =& \frac{C}{{n{h_{n}}}}\biggl\Vert {K \biggl( {\frac{{{Y_{1}} - y}}{{{h_{n}}}}} \biggr) \frac{\alpha}{{G ( {{Y_{1}}} )}}} \biggr\Vert _{2 + \delta }^{2}\sum _{1 \le i < j \le k} {\sum_{s = {k_{i}}}^{{k_{i}} + p - 1} {\sum_{t = {k_{j}}}^{{k_{j}} + p - 1} { \bigl( {\alpha ( {t - s} )} \bigr)^{\delta / ( 2 + \delta)} } } }, \end{aligned}$$

now using the notation $u ( n ) := \sum_{j = n}^{\infty}{ ( {\alpha ( {j} )} )^{\frac{\delta }{{\delta + 2}}}}$, which is defined before, and A1 we get the following result:

4.5 $$\begin{aligned} \bigl\vert \mathrm{II}' \bigr\vert \leq& \frac{C}{{n{h_{n}}}} \biggl\Vert {K \biggl( {\frac{{{Y_{1}} - y}}{{{h_{n}}}}} \biggr)\frac{\alpha}{{G ( {{Y_{1}}} )}}} \biggr\Vert _{2 + \delta}^{2}\sum_{1 \le i < j \le k} \sum _{s = {k_{i}}}^{{k_{i}} + p - 1} {\sum _{t = {k_{j}}}^{{k_{j}} + p - 1} { \bigl( {\alpha ( {t - s} )} \bigr)^{\delta / ( 2 + \delta) } } } \\ \le& \frac{Ckp}{{nh_{n}^{\delta/ ( 2 + \delta )} }}{ \biggl[ { \int_{ - 1}^{1} {{{\bigl\vert {K ( t )} \bigr\vert }^{2 + \delta }}\frac{{f ( {y + t{h_{n}}} )}}{{{G^{1 + \delta}} ( {y + t{h_{n}}} )}}\,dt} } \biggr]^{\delta / ( 2 + \delta)} } \sum _{j = q}^{\infty}{ \bigl( {\alpha ( {j} )} \bigr)^{\delta / ( 2 + \delta )} } \\ =& O \bigl( h_{n}^{ - \delta / 2 + \delta}u ( q ) \bigr). \end{aligned}$$

Under Assumption A2 we can write

4.6 $$\begin{aligned} \bigl\vert {\mathrm{III}'} \bigr\vert \le & 2 \sum_{m = 1}^{k} {\sum _{{k_{m}} \le i < j \le{k_{m}} + p - 1} {\bigl\vert {\mathit {Cov}( {{W_{ni}},{W_{nj}}} )} \bigr\vert } } \\ \le & \frac{2}{{n{h_{n}}}}\sum_{m = 1}^{k} { \sum_{{k_{m}} \le i < j \le{k_{m}} + p - 1} { \biggl\{ {E\biggl\vert {K \biggl( { \frac{{{Y_{i}} - y}}{{{h_{n}}}}} \biggr) K \biggl( {\frac{{{Y_{j}} - y}}{{{h_{n}}}}} \biggr) \frac {{{\alpha^{2}}}}{{G ( {{Y_{i}}} )G ( {{Y_{j}}} )}}} \biggr\vert } } } \\ & {}+ {{E^{2}} \biggl[ {K \biggl( {\frac{{{Y_{i}} - y}}{{{h_{n}}}}} \biggr) \frac{\alpha}{{G ( {{Y_{i}}} )}}} \biggr]} \biggr\} \\ \le & \frac{2}{{n{h_{n}}}}\sum_{m = 1}^{k} \sum_{{k_{m}} \le i < j \le{k_{m}} + p - 1} \biggl\{ \int \int\biggl\vert {K \biggl( {\frac {{{u_{1}} - y}}{{{h_{n}}}}} \biggr) K \biggl( { \frac{{{u_{2}} - y}}{{{h_{n}}}}} \biggr)} \biggr\vert \\ & {}\times{f^{*}} ( {{u_{2}}|{u_{1}}} ){f^{*}} ( {{u_{1}}} )\,d{u_{1}}\,d{u_{2}} + \biggl( { \int{K \biggl( {\frac{{u - y}}{{{h_{n}}}}} \biggr)f ( u )\,du} } \biggr)^{2} \biggr\} \\ \le & \frac{{C{h_{n}}}}{n}\sum_{m = 1}^{k} \sum_{{k_{m}} \le i < j \le{k_{m}} + p - 1} \biggl\{ \int_{ - 1}^{1} \int_{ - 1}^{1} \bigl\vert {K ( s )K ( t )} \bigr\vert \,ds \,dt + \\ & {}+ \biggl[ { \int_{ - 1}^{1} {K ( t )f ( {y + t{h_{n}}} ) \,dt} } \biggr]^{2} \biggr\} \\ \le & C\frac{{k{p^{2}}{h_{n}}}}{n} \biggl\{ { \int_{ - 1}^{1} { \int_{ - 1}^{1} {\bigl\vert {K ( s )K ( t )} \bigr\vert \,ds\,dt} } + {{ \biggl[ { \int_{ - 1}^{1} {K ( t )f ( {y + t{h_{n}}} ) \,dt} } \biggr]}^{2}}} \biggr\} \\ = & O ( ph_{n} ). \end{aligned}$$

Now, using (4.4), (4.5), (4.6), and (4.2), we have

4.7 $$\begin{aligned}& \mathit {Var}\bigl( {{{\mathcal {J}}' _{n}}} \bigr) = O \biggl( {\frac{{kp}}{n} + h_{n}^{ - \delta/ 2 + \delta} u ( q ) + ph_{n}} \biggr) \\& \hphantom{ \mathit {Var}\bigl( {{{\mathcal {J}}' _{n}}} \bigr)}= O ( 1 ), \end{aligned}$$

4.8 $$\begin{aligned}& \mathit {Var}\bigl( {{{\mathcal {J}}'' _{n}}} \bigr) = \mathit {Var}\Biggl( {\sum_{m = 1}^{k} {{j''_{nm}}} } \Biggr) \\& \hphantom{ \mathit {Var}\bigl( {{{\mathcal {J}}'' _{n}}} \bigr) }= \sum_{m = 1}^{k} {\sum _{i = {l_{m}}}^{{l_{m}} + q - 1} {\mathit {Var}( {{W_{ni}}} )} } + 2\sum _{1 \le i < j \le k} {\mathit {Cov}\bigl( {{j''_{ni}},{j''_{nj}}} \bigr)} \\& \hphantom{ \mathit {Var}\bigl( {{{\mathcal {J}}'' _{n}}} \bigr) }\quad {} + 2\sum_{m = 1}^{k} {\sum _{{l_{m}} \le i < j \le{l_{m}} + q - 1} {\mathit {Cov}( {{W_{ni}},{W_{nj}}} )} } \\& \hphantom{ \mathit {Var}\bigl( {{{\mathcal {J}}'' _{n}}} \bigr) }=: \mathrm{I}'' + \mathrm{II}'' + \mathrm{III}''. \end{aligned}$$

By the same argument as is used for $\vert {\mathrm{I}'} \vert $ and $\vert {\mathrm{II}'} \vert $ and $\vert {\mathrm{III}'} \vert $, it can be concluded that

4.9 $$ \begin{aligned} &\bigl\vert {\mathrm{I}''} \bigr\vert = O \biggl( {\frac{{kq}}{n}} \biggr), \\ &\bigl\vert {\mathrm{II}''} \bigr\vert =O \bigl( {h_{n}^{ - \delta/ ( 2 + \delta)} u ( q )} \bigr), \\ &\bigl\vert {\mathrm{III}''} \bigr\vert = O ( {q{h_{n}}} ). \end{aligned} $$

Now, using (4.8) and (4.9), we have

4.10 $$\begin{aligned} \mathit {Var}\bigl( {{{\mathcal {J}}'' _{n}}} \bigr) =& O \biggl( {\frac{{kq}}{n} + {h_{n}^{ - \delta/ ( 2 + \delta)} u ( q )} + q{h_{n}}} \biggr) \\ =& O \bigl( \lambda''_{n} \bigr). \end{aligned}$$

Similarly

4.11 $$\begin{aligned} \mathit {Var}\bigl( {{{\mathcal {J}}''' _{n}}} \bigr) =& \mathit {Var}\Biggl( {\sum_{i = k ( {p + q} ) + 1}^{n} {{W_{ni}}} } \Biggr) \\ =& \sum_{i = k ( {p + q} ) + 1}^{n} {\mathit {Var}( {{W_{ni}}} )} + 2\sum_{k ( {p + q} ) + 1 \le i < j \le n} {\mathit {Cov}( {{W_{ni}},{W_{nj}}} )} \\ =:& \mathrm{I}''' + \mathrm{II}''', \end{aligned}$$

and

4.12 $$ \begin{aligned} &\bigl\vert {\mathrm{I}'''} \bigr\vert = O \biggl( {\frac{1}{n} \bigl( {n - k ( {p + q} )} \bigr)} \biggr), \\ &\bigl\vert {\mathrm{II}'''} \bigr\vert = O \biggl( {\frac{{{p^{2}}{h_{n}}}}{n}} \biggr). \end{aligned} $$

So we can write

4.13 $$\begin{aligned} \mathit {Var}\bigl( {{{\mathcal {J}}''' _{n}}} \bigr) =& O \biggl( {\frac{1}{n} \bigl( { \bigl( {n - k ( {p + q} )} \bigr) + {p^{2}} {h_{n}}} \bigr)} \biggr) \\ =& O \biggl( {\frac{p}{n} ( {p{h_{n}} + 1} )} \biggr) \\ =& O \bigl( \lambda''' _{n} \bigr). \end{aligned}$$

Gathering all that is obtained above,

4.14 $$\begin{aligned} \bigl\vert {\sigma_{n}^{2} ( y ) - { \sigma^{2}} ( y )} \bigr\vert =& \Biggl\vert {\sum _{i = 1}^{n} {\mathit {Var}( {{W_{ni}}} )} - { \sigma^{2}} ( y )} \\ &{}+ 2\sum_{1 \le i < j \le k} {\mathit {Cov}\bigl( {{j'_{ni}},{j'_{nj}}} \bigr)} + 2\sum_{m = 1}^{k} {\sum _{{k_{m}} \le i < j \le {k_{m}} + p - 1} {\mathit {Cov}( {{W_{ni}},{W_{nj}}} )} } \\ &{}+ 2\sum_{1 \le i < j \le k} {\mathit {Cov}\bigl( {{j''_{ni}},{j''_{nj}}} \bigr)} + 2\sum_{m = 1}^{k} {\sum _{{l_{m}} \le i < j \le{l_{m}} + p - 1} {\mathit {Cov}( {{W_{ni}},{W_{nj}}} )} } \\ &{}+ 2\sum_{k ( {p + q} ) + 1 \le i < j \le n} {\mathit {Cov}( {{W_{ni}},{W_{nj}}} )} + 2\mathit {Cov}\bigl( {{{\mathcal {J}}' _{n}},{{\mathcal {J}}'' _{n}}} \bigr) \\ &{} + 2\mathit {Cov}\bigl( {{{\mathcal {J}}' _{n}},{{\mathcal {J}}''' _{n}}} \bigr) + 2\mathit {Cov}\bigl( {{{\mathcal {J}}'' _{n}},{{\mathcal {J}}''' _{n}}} \bigr) \Biggr\vert , \end{aligned}$$

and by letting

$$\begin{aligned} {A_{n}} :=& \sum_{1 \le i < j \le k} {\mathit {Cov}\bigl( {{j'_{ni}},{j'_{nj}}} \bigr)} + \sum_{m = 1}^{k} {\sum _{{k_{m}} \le i < j \le{k_{m}} + p - 1} {\mathit {Cov}( {{W_{ni}},{W_{nj}}} )} } \\ & {}+\sum_{1 \le i < j \le k} {\mathit {Cov}\bigl( {{j''_{ni}},{j''_{nj}}} \bigr)} + \sum_{m = 1}^{k} {\sum _{{l_{m}} \le i < j \le{l_{m}} + p - 1} {\mathit {Cov}( {{W_{ni}},{W_{nj}}} )} } \\ &{} + \sum_{k ( {p + q} ) + 1 \le i < j \le n} {\mathit {Cov}( {{W_{ni}},{W_{nj}}} )} + \mathit {Cov}\bigl( {{{\mathcal {J}}' _{n}},{{\mathcal {J}}'' _{n}}} \bigr) \\ &{} + \mathit {Cov}\bigl( {{{\mathcal {J}}' _{n}},{{\mathcal {J}}''' _{n}}} \bigr) + \mathit {Cov}\bigl( {{{\mathcal {J}}'' _{n}},{{\mathcal {J}}''' _{n}}} \bigr), \end{aligned}$$

we have

4.15 $$\begin{aligned} (4.14)\le\Biggl\vert {\sum_{i = 1}^{n} {\mathit {Var}( {{W_{ni}}} )} - {\sigma^{2}} ( y )} \Biggr\vert + 2 \vert {{A_{n}}} \vert . \end{aligned}$$

On the other hand using (4.7), (4.10), and (4.13), we have

4.16 $$ \begin{aligned} &\mathit {Cov}\bigl( {{{\mathcal {J}}' _{n}},{{\mathcal {J}}'' _{n}}} \bigr) \le { \bigl[ {\mathit {Var}\bigl( {{{\mathcal {J}}' _{n}}} \bigr)\mathit {Var}\bigl( {\mathcal {J}}'' _{n} \bigr)} \bigr]^{\frac{1}{2}}} \\ &\hphantom{\mathit {Cov}\bigl( {{{\mathcal {J}}' _{n}},{{\mathcal {J}}'' _{n}}} \bigr)}= O \bigl( {{\lambda_{n}^{\prime\prime1/2} }} \bigr), \\ &\mathit {Cov}\bigl( {{{\mathcal {J}}' _{n}},{{\mathcal {J}}''' _{n}}} \bigr) = O \bigl( {{\lambda_{n}^{\prime\prime\prime1/2} }} \bigr), \\ &\mathit {Cov}\bigl( {{{\mathcal {J}}'' _{n}},{{\mathcal {J}}''' _{n}}} \bigr) = O \bigl( {\lambda_{n}^{\prime\prime1/2} \lambda_{n}^{\prime\prime\prime1/2}} \bigr). \end{aligned} $$

So for $A_{n}$ we can write

4.17 $$\begin{aligned} \vert {{A_{n}}} \vert =& O \biggl( { h_{n}^{ - \delta/ 2 + \delta} u ( q ) + p{h_{n}} + q{h_{n}} + {p^{2}} \frac{{{h_{n}}}}{n} + \lambda_{n}^{\prime\prime 1/2} + \lambda_{n}^{\prime\prime\prime1/2} + \lambda_{n}^{\prime\prime1/2} \lambda_{n}^{\prime\prime\prime1/2} } \biggr) \\ =& O \bigl( { h_{n}^{ - \delta/ 2 + \delta} u ( q ) + p{h_{n}} + q{h_{n}} + \lambda_{n}^{\prime\prime1/2} + \lambda_{n}^{\prime\prime\prime1/2} + \lambda_{n}^{\prime\prime1/2}\lambda_{n}^{\prime\prime\prime1/2}} \bigr). \end{aligned}$$

On the other hand from (4.3), it can easily be concluded that $\sum_{i = 1}^{n} {\mathit {Var}( {{W_{ni}}} )} \to{\sigma ^{2}} ( y )$ as $n \to\infty$. Now under Assumptions A1 and A4 $\vert {{A_{n}}} \vert \to0$, so $\sigma_{n}^{2} ( y ) \to {\sigma^{2}} ( y )$. If *f* and *G* have bounded first derivatives in a neighborhood of *y*, we can write

4.18 $$\begin{aligned}& \Biggl\vert {\sum_{i = 1}^{n} {\mathit {Var}( {{W_{ni}}} )} - {\sigma^{2}} ( y )} \Biggr\vert \\& \quad = \biggl\vert {\frac{1}{{{h_{n}}}} \biggl\{ {E \biggl[ {{K^{2}} \biggl( {\frac {{{Y_{1}} - y}}{{{h_{n}}}}} \biggr)\frac{{{\alpha^{2}}}}{{{G^{2}} ( {{Y_{1}}} )}}} \biggr] - {E^{2}} \biggl[ {K \biggl( {\frac{{{Y_{1}} - y}}{{{h_{n}}}}} \biggr) \frac{\alpha}{{G ( {{Y_{1}}} )}}} \biggr]} \biggr\} - {\sigma^{2}} ( y )} \biggr\vert \\& \quad = \biggl\vert {\frac{1}{{{h_{n}}}} \biggl\{ { \int{{K^{2}} \biggl( {\frac{{u - y}}{{{h_{n}}}}} \biggr) \frac{{\alpha f ( u )}}{{G ( u )}}\,du} - {{ \biggl[ { \int{K \biggl( {\frac{{u - y}}{{{h_{n}}}}} \biggr)f ( u )\,du} } \biggr]}^{2}}} \biggr\} - {\sigma^{2}} ( y )} \biggr\vert \\& \quad = \biggl\vert { \int_{ - 1}^{1} {{K^{2}} ( t ) \frac{{G ( y ) [ {f ( {y + t{h_{n}}} ) - f ( y )} ] + f ( y ) [ {G ( {y + t{h_{n}}} ) - G ( y )} ]}}{{G ( {y + {h_{n}}t} )G ( y )}}\,dt} } \\& \quad\quad{} - {h_{n}} {{ \biggl[ { \int_{ - 1}^{1} {K ( t )f ( {y + t{h_{n}}} ) \,dt} } \biggr]}^{2}} \biggr\vert \\& \quad = O ( {{h_{n}}} ). \end{aligned}$$

From (4.14) we get the following result:

4.19 $$\begin{aligned}& \bigl\vert {\sigma_{n}^{2} ( y ) - { \sigma^{2}} ( y )} \bigr\vert \\& \quad = O \bigl( {{h_{n}} + h_{n}^{ - \delta/ 2 + \delta} u ( q ) + p{h_{n}} + q{h_{n}} + \lambda_{n}^{\prime\prime1/2} + \lambda_{n}^{\prime\prime\prime1/2} + \lambda_{n}^{\prime\prime1/2}\lambda_{n}^{\prime\prime\prime1/2}} \bigr) \\& \quad = O \bigl( {{h_{n}} + h_{n}^{ - \delta/ 2 + \delta} u ( q ) + p{h_{n}} + \lambda_{n}^{\prime\prime1/2} + \lambda_{n}^{\prime\prime\prime1/2} + \lambda_{n}^{\prime\prime1/2}\lambda_{n}^{\prime\prime\prime1/2}} \bigr) \\& \quad = O \bigl( {{h_{n}} ( p + 1 ) + h_{n}^{ - \delta/ 2 + \delta} u ( q ) + \lambda _{n}^{\prime\prime1/2} + \lambda_{n}^{\prime\prime\prime1/2} + \lambda_{n}^{\prime\prime1/2}\lambda_{n}^{\prime\prime\prime1/2}} \bigr) , \end{aligned}$$

and the proof is completed. □

Before starting the next lemma, we note that

4.20 $$\begin{aligned}& \frac{{\sqrt{n{h_{n}}} [ {{f_{n}} ( y ) - E{f_{n}} ( y )} ]}}{{{\sigma_{n}} ( y )}} \\& \quad = \frac{1}{{{\sigma_{n}} ( y )\sqrt{n{h_{n}}} }}\sum_{i = 1}^{n} { \biggl\{ {K \biggl( {\frac{{{Y_{i}} - y}}{{{h_{n}}}}} \biggr)\frac {\alpha}{{G ( {{Y_{i}}} )}} - E \biggl[ {K \biggl( {\frac{{{Y_{i}} - y}}{{{h_{n}}}}} \biggr)\frac{\alpha}{{G ( {{Y_{i}}} )}}} \biggr]} \biggr\} } \\& \quad =: \sum_{i = 1}^{n} {{Z_{ni}}}. \end{aligned}$$

If we let $\sum_{i = 1}^{n} {{Z_{ni}}} =: {S_{n}}$, it can be observed that

4.21 $$\begin{aligned} S_{n} = {S'_{n}} + {S''_{n}} + {S'''_{n}}, \end{aligned}$$

in which

$$\begin{aligned}& {S'_{n}} = \sum_{m = 1}^{k} {{Y'_{nm}}} , \quad\quad {Y'_{nm}} = \sum_{i = {k_{m}}}^{{k_{m}} + p - 1} {{Z_{ni}}}, \\& {S''_{n}} = \sum _{m = 1}^{k} {{Y''_{nm}}} , \quad\quad {Y''_{nm}} = \sum _{i = {l_{m}}}^{{l_{m}} + q - 1} {{Z_{ni}}}, \\& S''' = \sum _{m = 1}^{k} {{Y'''_{nm}}} , \quad\quad {Y'''_{nm}} = \sum _{i = k ( {p + q} ) + 1}^{n} {{Z_{ni}}}. \end{aligned}$$

### Lemma 3

*Suppose that Assumptions* A1-A3(i) *and* A4 *are satisfied and* *f* *and* *G* *are continuous in a neighborhood of* *y* *for* $y\geq a_{F}$. *Then for such* *y’s we have*

$$\begin{aligned}& \mathrm{P} \bigl( {\bigl\vert {{S''_{n}}} \bigr\vert > \lambda_{n}^{\prime\prime\frac{{1 }}{3}}} \bigr) = O \bigl( {\lambda _{n}^{\prime\prime\frac{{1 }}{3}} } \bigr), \\& \mathrm{P} \bigl( {\bigl\vert {{S'''_{n}}} \bigr\vert > \lambda_{n}^{\prime\prime\prime\frac{{1 }}{3}}} \bigr) = O \bigl( {\lambda _{n}^{\prime\prime\prime\frac{{1}}{3}} } \bigr). \end{aligned}$$

### Proof

With the aid of Lemma 2 we can write

4.22 $$\begin{aligned} E{ \bigl( {{S''_{n}}} \bigr)^{2}} =& \frac{1}{{\sigma_{n}^{2} ( y )}}E{ \bigl[ {{{\mathcal {J}}'' _{n}} - E \bigl( {{{\mathcal {J}}'' _{n}}} \bigr)} \bigr]^{2}} \\ =& O \bigl( {{\lambda''_{n}}} \bigr). \end{aligned}$$

The same argument shows that $E{ ( {{S'''_{n}}} )^{2}} = O ( {{\lambda'''_{n}}} )$, so we have

4.23 $$\begin{aligned} \mathrm{P} \bigl( {\bigl\vert {{S''_{n}}} \bigr\vert > \lambda _{n}^{\prime\prime\frac{{1}}{3}}} \bigr) \le& \frac{{E{{ ( {{S''_{n}}} )}^{2}}}}{{\lambda_{n}^{\prime\prime\frac{2}{3}}}} \\ =& O \bigl( {\lambda_{n}^{\prime\prime\frac{1}{3}} } \bigr) \end{aligned}$$

and

4.24 $$\begin{aligned} \mathrm{P} \bigl( {\bigl\vert {{S'''_{n}}} \bigr\vert > \lambda_{n}^{\prime\prime\prime\frac{{1}}{3}}} \bigr) \le& \frac{{E{{ ( {{S'''_{n}}} )}^{2}}}}{{\lambda_{n}^{\prime\prime\prime\frac{2}{3}}}} \\ =& O \bigl( {\lambda_{n}^{\prime\prime\prime\frac{1}{3}} } \bigr). \end{aligned}$$

So the proof is completed. □

In the following let ${H_{n}}: = \sum_{m = 1}^{k} {{X_{nm}}} $ in which ${{X_{nm}}}$, $m = 1,\ldots,k$, are independent random variables with the same distribution as ${Y'_{nm}}$, $m = 1,\ldots,k$. *φ* and ${\varphi'}$ are, respectively, the characteristic functions of $S'_{n}$ and $H_{n}$. Also let $s^{\prime 2}_{n}: = \sum_{m = 1}^{k} {\mathit {Var}( {{X_{nm}}} )}$ and $s^{2}_{n}: = \sum_{m = 1}^{k} {\mathit {Var}( {{Y'_{nm}}} )} $.

### Lemma 4

*Under the assumptions of Lemma* 3, *for* $y\geq a_{F}$ *we have the following*:

$$\bigl\vert {s_{n}^{2} - 1} \bigr\vert = O \bigl( \lambda_{n}^{\prime\prime1/2} + \lambda_{n}^{\prime\prime\prime1/2} +h_{n}^{ - \delta/ ( 2 + \delta)} u ( q ) \bigr). $$

### Proof

It can easily be seen that $s_{n}^{2} = E{ ( {{S'_{n}}} )^{2}} - 2\sum_{1 \le i < j \le k} {\mathit {Cov}( {{Y'_{ni}},{Y'_{nj}}} )} $, $E ( {S_{n}^{2}} ) = 1$ and

4.25 $$\begin{aligned} \bigl\vert {E{{ \bigl( {{S'_{n}}} \bigr)}^{2}} - 1} \bigr\vert =& \bigl\vert {E{{ \bigl( {{S'_{n}}} \bigr)}^{2}} - E \bigl( {S_{n}^{2}} \bigr)} \bigr\vert \\ =& \bigl\vert {E{{ \bigl( {{S'_{n}}} \bigr)}^{2}} - E{{ \bigl( {{S'_{n}} + {S''_{n}} + {S'''_{n}}} \bigr)}^{2}}} \bigr\vert \\ =& \bigl\vert E \bigl( S''_{n} + S'''_{n} \bigr)^{2} - 2E \bigl( S'_{n} \bigl( S''_{n} + S'''_{n} \bigr) \bigr) \bigr\vert . \end{aligned}$$

Using (4.25) and Lemma 2, we can write

4.26 $$\begin{aligned} \bigl\vert {s_{n}^{2} - 1} \bigr\vert =& \biggl\vert {E{{ \bigl( {{S'_{n}}} \bigr)}^{2}} - 2\sum_{1 \le i < j \le k} {\mathit {Cov}\bigl( {{Y'_{ni}},{Y'_{nj}}} \bigr)} - 1} \biggr\vert \\ \le& \bigl\vert {E{{ \bigl( {{S'_{n}}} \bigr)}^{2}} - 1} \bigr\vert + 2\biggl\vert {\sum _{1 \le i < j \le k} {\mathit {Cov}\bigl( {{Y'_{ni}},{Y'_{nj}}} \bigr)} } \biggr\vert \\ =& \bigl\vert {E{{ \bigl( {{S''_{n}} + {S'''_{n}}} \bigr)}^{2}} - 2E \bigl( {{S'_{n}} \bigl( {{S''_{n}} + {S'''_{n}}} \bigr)} \bigr)} \bigr\vert + 2\biggl\vert {\sum_{1 \le i < j \le k} {\mathit {Cov}\bigl( {{Y'_{ni}},{Y'_{nj}}} \bigr)} } \biggr\vert \\ =& O \bigl( {\lambda_{n}^{\prime\prime1/2} + \lambda_{n}^{\prime\prime\prime1/2}} \bigr) + 2\biggl\vert {\sum_{1 \le i < j \le k} {\mathit {Cov}\bigl( {{j'_{ni}},{j'_{nj}}} \bigr)} } \biggr\vert . \end{aligned}$$

On the other hand, from Lemma 2 we know that $\sum_{1 \le i < j \le k} {\mathit {Cov}( {{j'_{ni}},{j'_{nj}}} )} = O ( h_{n}^{ - \delta/ ( 2 + \delta )} u ( q ) )$, so substituting this in (4.26), gives the result,

$$\bigl\vert {s^{\prime 2}_{n} - 1} \bigr\vert = O \bigl( {\lambda_{n}^{\prime\prime1/2} + \lambda_{n}^{\prime\prime\prime1/2} + h_{n}^{ - \delta/ ( 2 + \delta)} u ( q )} \bigr). $$

 □

### Lemma 5

[18]

*Let* $\{ {{X_{j}},j \ge1} \}$ *be a stationary sequence with mixing coefficient* $\alpha ( k )$ *and suppose that* $E (X_{n} )=0$, $r>2$, *and there exist* $\tau> 0$ *and* $\lambda> \frac{{r ( {r + \tau} )}}{{2\tau}}$ *such that* $\alpha ( n ) = O ( {{n^{ - \lambda}}} )$ *and also* $E{\vert {{X_{i}}} \vert ^{r + \tau}} < \infty$. *In this case*, *for any* $\epsilon> 0$, *there exists a constant* *C*, *for which we have*

$$E{\Biggl\vert {\sum_{i = 1}^{n} {{X_{i}}} } \Biggr\vert ^{r}} \le C \Biggl[ {{n^{\varepsilon}}\sum_{i = 1}^{n} {E{{ \vert {{X_{i}}} \vert }^{r}}} + {{ \Biggl( {\sum _{i = 1}^{n} {\Vert {{X_{i}}} \Vert _{r + \tau }^{2}} } \Biggr)}^{r/2}}} \Biggr]. $$

### Lemma 6

*Under the assumptions of Lemma* 3 *for* $y\geq a_{F}$ *we have*

$$\sup_{x \in\mathbb{R}} \biggl\vert {\mathrm{P} \biggl( { \frac{{{H_{n}}}}{{{s_{n}}}} \le x} \biggr) - \Phi ( x )} \biggr\vert = O \biggl( h_{n}^{\frac{{2 ( {1 + \delta'} )}}{{2 + \delta}}}{ \biggl( {\frac{p}{{n{h_{n}}}}} \biggr)^{1 + \delta'}} \biggr). $$

### Proof

Using [19], Theorem 5.7, for $r>2$ we can write

4.27 $$ \sup_{x \in\mathbb{R}} \biggl\vert {\mathrm{P} \biggl( { \frac{{{H_{n}}}}{{{s_{n}}}} \le x} \biggr) - \Phi ( x )} \biggr\vert \le \frac{{C\sum_{m = 1}^{k} {E{{\vert {{X _{nm}}} \vert }^{r}}} }}{{s_{{n}}^{r}}}. $$

On the other hand, using Lemma 5 there exists $\tau> 0$ such that for any $\epsilon> 0$

4.28 $$\begin{aligned} \sum_{m = 1}^{k} {E{{\vert {{X _{nm}}} \vert }^{r}}} =& \sum _{m = 1}^{k} {E{{\vert {{Y_{nm}}} \vert }^{r}}} \\ =& \sum_{m = 1}^{k} {E{{\Biggl\vert {\sum _{i = {k_{m}}}^{{k_{m}} + p - 1} {{Z_{ni}}} } \Biggr\vert }^{r}}} \\ \le& \sum_{m = 1}^{k} {C \Biggl[ {{p^{\varepsilon}}\sum_{i = {k_{m}}}^{{k_{m}} + p - 1} {E{{ \vert {{Z_{ni}}} \vert }^{r}}} + {{ \Biggl( {\sum _{i = {k_{m}}}^{{k_{m}} + p - 1} {\Vert {{Z_{ni}}} \Vert _{r + \tau}^{2}} } \Biggr)}^{\frac{r}{2}}}} \Biggr]}. \end{aligned}$$

Let $\epsilon=\delta' $, $r=2+2\delta'$ for $0<2\delta'<\delta$ and $\tau=\delta-2\delta'$ and $\lambda> \frac{{ ( {1 + \delta'} ) ( {2 + \delta} )}}{{\delta- 2\delta'}}$, so we have

4.29 $$\begin{aligned} (4.28) =& C\sum_{m = 1}^{k} \biggl\{ \frac{{{p^{1 + \delta'}}}}{{{{ ( {n{h_{n}}} )}^{1 + \delta'}}}} \int{{{ \biggl[ {K \biggl( {\frac{{u - y}}{{{h_{n}}}}} \biggr)} \biggr]}^{2 ( {1 + \delta'} )}}\frac{{f ( u )}}{{{G^{1 + 2\delta'}} ( u )}}\,du} \\ & {} + \frac{{{p^{1 + \delta'}}}}{{{{ ( {n{h_{n}}} )}^{1 + \delta'}}}}{{ \biggl[ { \int{{{ \biggl[ {K \biggl( {\frac{{u - y}}{{{h_{n}}}}} \biggr)} \biggr]}^{2 + \delta}}\frac{{f ( u )}}{{{G^{1 + \delta'}} ( u )}}\,du} } \biggr]}^{\frac{{2 ( {1 + \delta'} )}}{{ ( {2 + \delta} )}}}} \biggr\} \\ =& C\sum_{m = 1}^{k} \biggl\{ \frac{{{p^{1 + \delta'}}}}{{{n^{1 + \delta'}}h_{n}^{\delta'}}} \int_{ - 1}^{1} {{{ \bigl[ {K ( t )} \bigr]}^{2 + \delta'}}\frac{{f ( {y + {h_{n}}t} )}}{{{G^{1 + \delta'}} ( {y + {h_{n}}t} )}}\,dt} \\ &{} + \frac{{{p^{1 + \delta'}}}}{{{n^{1 + \delta'}}h_{n}^{\frac {{\delta ( {1 + \delta'} )}}{{2 + \delta}}}}}{{ \biggl[ { \int _{ - 1}^{1} {{{ \bigl[ {K ( t )} \bigr]}^{2 + \delta}}\frac {{f ( {y + {h_{n}}t} )}}{{{G^{1 + \delta}} ( {y + {h_{n}}t} )}}\,dt} } \biggr]}^{\frac{{2 ( {1 + \delta'} )}}{{ ( {2 + \delta} )}}}} \biggr\} \\ \le& Ck \bigl[ {{p^{1 + \delta'}} {{ ( {n{h_{n}}} )}^{ - ( {1 + \delta'} )}} {h_{n}} + {p^{1 + \delta'}} {{ ( {n{h_{n}}} )}^{ - ( {1 + \delta'} )}}h_{n}^{\frac{{2 ( {1 + \delta'} )}}{{2 + \delta}}}} \bigr] \\ =& O \biggl( {h_{n}^{\frac{{2 ( {1 + \delta'} )}}{{2 + \delta }}}{{ \biggl( {\frac{p}{{n{h_{n}}}}} \biggr)}^{1 + \delta'}}} \biggr). \end{aligned}$$

From Lemma 4, $s_{n}^{2} \to1$, so the proof is completed. □

### Lemma 7

[20]

*Let* $\{ {{X_{j}},j \ge1} \}$ *be a stationary sequence with mixing coefficient* $\alpha ( k )$. *Suppose that* *p* *and* *q* *are positive integers*. *Let* ${T _{l}} = \sum_{j = ( {l - 1} ) ( {p + q} ) + 1}^{ ( {l - 1} ) ( {p + q} ) + p} {{X_{j}}} $ *in which* $1\leq l \leq k$. *If* $s,r>0$ *such that* ${s^{ - 1}} + {r^{ - 1}} = 1$, *there exists a constant* $C>0$ *such that*

$$\Biggl\vert {E\exp \Biggl( {it\sum_{l = 1}^{k} {{T _{l}}} } \Biggr) - \prod_{l = 1}^{k} {E\exp ( {it{T _{l}}} )} } \Biggr\vert \le C\vert t \vert { \alpha^{1/s}} ( q )\sum_{l = 1}^{k} {{{\Vert {{T _{l}}} \Vert }_{r}}}. $$

### Lemma 8

*Under the assumptions of Lemma* 3 *for* $y\geq a_{F}$ *we have*

$$\sup_{x \in\mathbb{R}} \bigl\vert {\mathrm{P} \bigl( {{S'_{n}} \le x} \bigr) - \mathrm{P} ( {{H_{n}} \le x} )} \bigr\vert = O \biggl( \bigl( {{\lambda '''_{n}}\alpha ( q )} \bigr)^{1/4} + h_{n}^{\frac{{2 ( {1 + \delta'} )}}{{2 + \delta}}} \biggl( { \frac{p}{{n{h_{n}}}}} \biggr)^{1 + \delta'} \biggr). $$

### Proof

By letting $b=1$ in [19], Theorem 5.3, p.147, for any $T>0$ we have

4.30 $$\begin{aligned} \sup_{x \in\mathbb{R}} \bigl\vert {\mathrm{ P} \bigl( {{S'_{n}} \le x} \bigr) - \mathrm{P} ( {{H_{n}} \le x} )} \bigr\vert \le& \int_{ - T}^{T} {\biggl\vert {\frac{{\varphi ( t ) - \varphi' ( t )}}{t}} \biggr\vert \,dt} \\ & {}+ T\sup_{x \in\mathbb{R}} \int_{\vert u \vert \le\frac{C}{T}} {\bigl\vert {\mathrm{P} ( {{H_{n}} \le u + x} ) - \mathrm{P} ( {{H_{n}} \le x} )} \bigr\vert \,du} \\ =:& {L_{n1}} + {L_{n2}}. \end{aligned}$$

Now by letting $s=r=2$ in Lemma 7, there exists a constant $C>0$ for which we have

4.31 $$\begin{aligned}& \begin{aligned}[b] \bigl\vert {\varphi ( t ) - \varphi' ( t )} \bigr\vert &= \Biggl\vert {E\exp \Biggl( {it\sum_{m = 1}^{k} {{Y_{nm}}} } \Biggr) - \prod_{m = 1}^{k} {E\exp ( {it{Y_{nm}}} )} } \Biggr\vert \\ &\le C\vert t \vert { \bigl( {\alpha ( q )} \bigr)^{\frac {1}{2}}}\sum _{m = 1}^{k} {{{\Vert {{Y_{nm}}} \Vert }_{2}}} \\ &= Ck\vert t \vert { \bigl( {\alpha ( q )} \bigr)^{\frac {1}{2}}} {E^{\frac{1}{2}}} {\Biggl\vert {\sum_{i = {k_{m}}}^{{k_{m}} + p - 1} {{Z_{ni}}} } \Biggr\vert ^{2}}, \end{aligned} \end{aligned}$$

4.32 $$\begin{aligned}& \begin{aligned}[b] E{ ( {{Z_{n1}}} )^{2}} &= \frac{{{\alpha^{2}}}}{{n{h_{n}}\sigma _{n}^{2} ( y )}}E{ \biggl\{ {K \biggl( {\frac{{{Y_{1}} - y}}{{{h_{n}}}}} \biggr) \frac{1}{{G ( {{Y_{1}}} )}} - E \biggl[ {K \biggl( {\frac{{{Y_{1}} - y}}{{{h_{n}}}}} \biggr) \frac{1}{{G ( {{Y_{1}}} )}}} \biggr]} \biggr\} ^{2}} \\ &\le \frac{1}{{n\sigma_{n}^{2} ( y )}} \biggl\{ { \int_{ - 1}^{1} {{K^{2}} ( t ) \frac{{f ( {y + t{h_{n}}} )}}{{G ( {y + t{h_{n}}} )}}\,dt} + {h_{n}} {{ \biggl[ { \int_{ - 1}^{1} {K ( t )f ( {y + t{h_{n}}} ) \,dt} } \biggr]}^{2}}} \biggr\} \\ &= O \bigl( {{n^{ - 1}}} \bigr), \end{aligned} \end{aligned}$$

4.33 $$\begin{aligned}& \begin{aligned}[b] E ( {{Z_{n1}} {Z_{n2}}} ) &\le \frac{1}{{n{h_{n}}\sigma _{n}^{2} ( y )}} \int_{ - 1}^{1} { \int_{ - 1}^{1} {\biggl\vert {K \biggl( { \frac{{{y_{1}} - u}}{{{h_{n}}}}} \biggr)K \biggl( {\frac{{{y_{2}} - u}}{{{h_{n}}}}} \biggr)} \biggr\vert {f^{*}} ( {{y_{2}}|{y_{1}}} ){f^{*}} ( {{y_{1}}} ) \,d{y_{1}}\,d{y_{2}}} } \\ &= O \biggl( {\frac{{{h_{n}}}}{n}} \biggr). \end{aligned} \end{aligned}$$

Now using (4.32) and (4.33) we have

4.34 $$\begin{aligned} E{\Biggl\vert {\sum_{i = {k_{m}}}^{{k_{m}} + p - 1} {{Z_{ni}}} } \Biggr\vert ^{2}} =& \sum _{i = {k_{m}}}^{{k_{m}} + p - 1} {E{{ ( {{Z_{ni}}} )}^{2}}} + 2\sum_{{k_{m}} \le i < j \le{k_{m}} + p - 1} {E{Z_{ni}} {Z_{nj}}} \\ =& O \biggl( {\frac{p}{n} ( {1 + p{h_{n}}} )} \biggr), \end{aligned}$$

so

4.35 $$\begin{aligned} {L_{n1}} =&O \biggl( {T{{ \biggl( {\frac{{p\alpha ( q )}}{n} ( {1 + p{h_{n}}} )} \biggr)}^{1/2}}} \biggr) \\ =& O \bigl( {T{{ \bigl( {{\lambda'''_{n}} \alpha ( q )} \bigr)}^{1/2}}} \bigr). \end{aligned}$$

On the other hand applying Lemma 6 gives

4.36 $$\begin{aligned}& \sup_{x \in\mathbb{R}} \bigl\vert {\mathrm{P} ( {{H_{n}} \le u + x} ) - \mathrm{P} ( {{H_{n}} \le x} )} \bigr\vert \\& \quad \le\sup_{x \in\mathbb{R}} \biggl\vert {\mathrm{ P} \biggl( { \frac{{{H_{n}}}}{{{s_{n}}}} \le\frac{{u + x}}{{{s_{n}}}}} \biggr) - \Phi \biggl( { \frac{{u + x}}{{{s_{n}}}}} \biggr)} \biggr\vert \\& \quad\quad{} + \sup_{x \in\mathbb{R}} \biggl\vert {\mathrm{ P} \biggl( { \frac{{{H_{n}}}}{{{s_{n}}}} \le\frac{x}{{{s_{n}}}}} \biggr) - \Phi \biggl( { \frac{x}{{{s_{n}}}}} \biggr)} \biggr\vert + \sup_{x \in\mathbb{R}} \biggl\vert {\Phi \biggl( {\frac{{u + x}}{{{s_{n}}}}} \biggr) - \Phi \biggl( { \frac{{x}}{{{s_{n}}}}} \biggr)} \biggr\vert \\& \quad = O \biggl( {h_{n}^{\frac{{2 ( {1 + \delta'} )}}{{2 + \delta }}}{{ \biggl( {\frac{p}{{n{h_{n}}}}} \biggr)}^{1 + \delta'}}} \biggr) + O \biggl( {\frac{{\vert u \vert }}{{{s_{n}}}}} \biggr), \end{aligned}$$

so

4.37 $$ {L_{n2}} = O \biggl( {h_{n}^{\frac{{2 ( {1 + \delta'} )}}{{2 + \delta}}}{{ \biggl( { \frac{p}{{n{h_{n}}}}} \biggr)}^{1 + \delta'}} + \frac {1}{T}} \biggr). $$

By choosing $T = { ( {\alpha ( q ){\lambda'''_{n}}} )^{-1/4}}$ we get the following result:

4.38 $$\begin{aligned}& \sup_{x \in\mathbb{R}} \bigl\vert {\mathrm{ P} \bigl( {{S'_{n}} \le x} \bigr) - \mathrm{P} ( {{H_{n}} \le x} )} \bigr\vert \\& \quad = O \biggl( h_{n}^{\frac{{2 ( {1 + \delta'} )}}{{2 + \delta }}}{ \biggl( {\frac{p}{{n{h_{n}}}}} \biggr)^{1 + \delta'}} + { \bigl( {{\lambda'''_{n}} \alpha ( q )} \bigr)^{1/4}} \biggr) \\& \quad = O ( b_{n} ), \end{aligned}$$

and the lemma is proved. □

### Lemma 9

[21]

*Let* *X* *and* *Y* *be random variables*. *For any* $a>0$ *we have*

$$\sup_{t \in\mathbb{R}} \bigl\vert {\mathrm{P} ( {X + Y \le t} ) - \Phi ( t )} \bigr\vert \le\sup_{t \in\mathbb{R}} \bigl\vert {\mathrm{P} ( {X \le t} ) - \Phi ( t )} \bigr\vert + \frac{a}{{\sqrt{2\pi} }} + \mathrm{P} \bigl( {\vert Y \vert > a} \bigr). $$

### Proof of Theorem 1

Using (4.21) and Lemma 9, for any $a_{1}>0$ and $a_{2}>0$ we can write

4.39 $$\begin{aligned}& \sup_{x \in\mathbb{R}} \bigl\vert {\mathrm{ P} \bigl[ { \sqrt{n{h_{n}}} \bigl( {{f_{n}} ( y ) - E{f_{n}} ( y )} \bigr) \le x{\sigma_{n}} ( y )} \bigr] - \Phi ( x )} \bigr\vert \\& \quad = \sup_{x \in\mathbb{R}} \bigl\vert {\mathrm{ P} \bigl( {{S'_{n}} + {S''_{n}} + {S'''_{n}} \le x} \bigr) - \Phi ( x )} \bigr\vert \\& \quad \le\sup_{x \in\mathbb{R}} \bigl\vert {\mathrm{ P } \bigl( {{S'_{n}} \le x} \bigr) - \Phi ( x )} \bigr\vert + \frac{{{a_{1}}}}{{\sqrt{2\pi} }} + \frac{{{a_{2}}}}{{\sqrt {2\pi} }} + \mathrm{ P} \bigl( {\bigl\vert {{S''_{n}}} \bigr\vert > {a_{1}}} \bigr) + \mathrm{P} \bigl( {\bigl\vert {{S'''_{n}}} \bigr\vert > {a_{1}}} \bigr). \end{aligned}$$

By choosing ${a_{1}} = {{\lambda''_{n}}^{1/3}}$ and ${a_{2}} = {\lambda _{n}^{\prime\prime\prime1/3}}$ and using Lemma 3, we have

4.40 $$ (4.39)= \sup_{x \in\mathbb{R}} \bigl\vert {\mathrm{P} \bigl( {{S'_{n}} \le x} \bigr) - \Phi ( x )} \bigr\vert + O \bigl( {{{\lambda''_{n}}^{1/3}} + {{\lambda '''_{n}}^{1/3}}} \bigr). $$

On the other hand using Lemmas 8, 4, and 6 we have

4.41 $$\begin{aligned}& \sup_{x \in\mathbb{R}} \bigl\vert {\mathrm{P} \bigl( {{S'_{n}} \le x} \bigr) - \Phi ( x )} \bigr\vert \\& \quad \le\sup_{x \in\mathbb{R}} \bigl\vert {\mathrm{P} \bigl( {{S'_{n}} \le x} \bigr) - \mathrm{P} ( {{H_{n}} \le x} )} \bigr\vert + \sup_{x \in\mathbb{R}} \bigl\vert {\mathrm{P} ( {{H_{n}} \le x} ) - \Phi ( x )} \bigr\vert \\& \quad \le\sup_{x \in\mathbb{R}} \bigl\vert {\mathrm{P} \bigl( {{S'_{n}} \le x} \bigr) - \mathrm{P} ( {{H_{n}} \le x} )} \bigr\vert + \sup_{x \in\mathbb{R}} \biggl\vert {\mathrm{P} ( {{H_{n}} \le x} ) - \Phi \biggl( { \frac{x}{{{s_{n}}}}} \biggr)} \biggr\vert \\& \quad\quad{} + \sup_{x \in\mathbb{R}} \biggl\vert {\Phi \biggl( { \frac{x}{{{s_{n}}}}} \biggr) - \Phi ( x )} \biggr\vert \\& \quad = O \biggl( {h_{n}^{\frac{{2 ( {1 + \delta'} )}}{{2 + \delta }}}{{ \biggl( {\frac{p}{{n{h_{n}}}}} \biggr)}^{1 + \delta'}} + {{ \bigl( {{\lambda'''_{n}} \alpha ( q )} \bigr)}^{1/4}}} \biggr) + O \biggl( {h_{n}^{\frac{{2 ( {1 + \delta'} )}}{{2 + \delta}}}{{ \biggl( {\frac{p}{{n{h_{n}}}}} \biggr)}^{1 + \delta'}}} \biggr) \\& \quad\quad{} + O \bigl( {\bigl\vert {s_{n}^{2} - 1} \bigr\vert } \bigr) \\& \quad = O \biggl( {h_{n}^{\frac{{2 ( {1 + \delta'} )}}{{2 + \delta }}}{{ \biggl( {\frac{p}{{n{h_{n}}}}} \biggr)}^{1 + \delta'}} + {{ \bigl( {{\lambda'''_{n}} \alpha ( q )} \bigr)}^{1/4}} + \lambda_{n}^{\prime\prime1/2} + \lambda_{n}^{\prime\prime\prime1/2} + h_{n}^{ - \delta/ ( 2 + \delta)} u ( q )} \biggr). \end{aligned}$$

So the proof is completed. □

### Proof of Theorem 2

According to Lemma 9 for any $a>0$ we can write

4.42 $$\begin{aligned}& \sup_{x \in\mathbb{R}} \bigl\vert {\mathrm{P} \bigl( {{{ ( {n{h_{n}}} )}^{1/2}} \bigl[ {{{\hat{f}}_{n}} ( y ) - E \bigl( {{f_{n}} ( y )} \bigr)} \bigr] \le x{\sigma_{n}} ( y )} \bigr) - \Phi ( x )} \bigr\vert \\& \quad \le\sup_{x \in\mathbb{R}} \bigl\vert {\mathrm{P} \bigl( {{{ ( {n{h_{n}}} )}^{1/2}} \bigl[ {{f_{n}} ( y ) - E \bigl( {{f_{n}} ( y )} \bigr)} \bigr] \le x{\sigma_{n}} ( y )} \bigr) - \Phi ( x )} \bigr\vert \\& \quad\quad{} + \frac{a}{{\sqrt{2\pi} }} + \mathrm{P} \biggl( {\frac{{\sqrt{n{h_{n}}} \vert {{{\hat{f}}_{n}} ( y ) - {f_{n}} ( y )} \vert }}{{{\sigma_{n}} ( y )}} > a} \biggr), \end{aligned}$$

and

4.43 $$\begin{aligned}& \mathrm{P} \biggl( {\frac{{\sqrt{n{h_{n}}} \vert {{{\hat{f}}_{n}} ( y ) - {f_{n}} ( y )} \vert }}{{{\sigma _{n}} ( y )}} > a} \biggr) \le\frac{1}{a}E \frac{{\sqrt {n{h_{n}}} \vert {{{\hat{f}}_{n}} ( y ) - {f_{n}} ( y )} \vert }}{{{\sigma_{n}} ( y )}}, \end{aligned}$$

4.44 $$\begin{aligned}& \begin{aligned}[b] & E\frac{{\sqrt{n{h_{n}}} \vert {{{\hat{f}}_{n}} ( y ) - {f_{n}} ( y )} \vert }}{{{\sigma_{n}} ( y )}} \\ &\quad \le\frac{1}{{\sqrt{n{h_{n}}} {\sigma_{n}} ( y )}}\sum_{i = 1}^{n} {E \biggl\vert {K \biggl( {\frac{{{Y_{i}} - y}}{{{h_{n}}}}} \biggr)\frac {{{\alpha_{n}}}}{{{G_{n}} ( {{Y_{i}}} )}} - K \biggl( {\frac{{{Y_{i}} - y}}{{{h_{n}}}}} \biggr)\frac{\alpha}{{G ( {{Y_{i}}} )}}} \biggr\vert } \\ &\quad = \frac{1}{{\sqrt{n{h_{n}}} {\sigma_{n}} ( y )}}\sum_{i = 1}^{n} E \biggl[ K \biggl( {\frac{{{Y_{i}} - y}}{{{h_{n}}}}} \biggr) \biggl\vert {\frac{{{\alpha_{n}}G ( {{Y_{i}}} ) - \alpha{G_{n}} ( {{Y_{i}}} )}}{{{G_{n}} ( {{Y_{i}}} )G ( {{Y_{i}}} )}}} \biggr\vert \biggr] \\ &\quad \le\frac{1}{\sqrt{n{h_{n}}} {\sigma_{n}} ( y )}\sum_{i = 1}^{n} E \biggl[ K \biggl( \frac{{Y_{i}} - y}{{h_{n}}} \biggr) \biggl\vert \frac{{G ( {{Y_{1}}} )\vert {{\alpha_{n}} - \alpha} \vert + \alpha \vert {{G_{n}} ( {{Y_{1}}} ) - G ( {{Y_{1}}} )} \vert }}{{G_{n}} ( {{Y_{i}}} )G ( {{Y_{i}}} )} \biggr\vert \biggr]. \end{aligned} \end{aligned}$$

From Lemma 5.2 of [16] we have

4.45 $$ \vert {{\alpha_{n}} - \alpha} \vert = O \biggl( { \sqrt{\frac{{\log \log n}}{n}} } \biggr) \quad \mbox{a.s.}, $$

and from [22] we have

4.46 $$ \sup_{y \ge{a_{F}}} \bigl\vert {{G_{n}} ( y ) - G ( y )} \bigr\vert = O \biggl( {\sqrt{\frac{{\log\log n}}{n}} } \biggr) \quad \mbox{a.s.} $$

So we can write

$$\begin{aligned} (4.44) \le& C\sqrt{n{h_{n}}} \biggl(\sqrt{ \frac{{\log\log n}}{n}} \biggr) \int_{ - 1}^{1} {\bigl\vert {K ( t )} \bigr\vert f ( {y + t{h_{n}}} )\,dt} \\ =& O ( {\sqrt{{h_{n}}\log\log n} } ). \end{aligned}$$

Now by choosing $a = { ( {{h_{n}}\log\log n} )^{1/4}}$ and using Theorem 1 we get the result

4.47 $$\begin{aligned}& \sup_{x \in\mathbb{R}} \bigl\vert {\mathrm{P} \bigl( {{{ ( {n{h_{n}}} )}^{1/2}} \bigl[ {{{\hat{f}}_{n}} ( y ) - E \bigl( {{f_{n}} ( y )} \bigr)} \bigr] \le x{\sigma_{n}} ( y )} \bigr) - \Phi ( x )} \bigr\vert \\& \quad = O \bigl( {{a_{n}} + {{ ( {{h_{n}}\log\log n} )}^{1/4}}} \bigr). \end{aligned}$$

 □

### Proof of Theorem 3

By the triangular inequality and using Lemma 1 for

$$a = \frac{{\sqrt{n{h_{n}}} \vert {E{f_{n}} ( y ) - f ( y )} \vert }}{{ \sigma ( y )}}, $$

we have

4.48 $$\begin{aligned}& \sup_{x \in\mathbb{R}} \bigl\vert {\mathrm{P} \bigl( { \sqrt{n{h_{n}}} \bigl[ {{{\hat{f}}_{n}} ( y ) - f ( y )} \bigr] \le x\sigma ( y )} \bigr) - \Phi ( x )} \bigr\vert \\& \quad \le\sup_{x \in\mathbb{R}} \biggl\vert {\mathrm{P} \biggl( { \sqrt{n{h_{n}}} \biggl[ {\frac{{{{\hat{f}}_{n}} ( y ) - f ( y )}}{{{\sigma_{n}} ( y )}}} \biggr] \le \frac{{\sigma ( y )}}{{{\sigma_{n}} ( y )}}x} \biggr) - \Phi \biggl( {\frac{{\sigma ( y )}}{{{\sigma _{n}} ( y )}}x} \biggr)} \biggr\vert \\& \quad\quad{} + \sup_{x \in\mathbb{R}} \biggl\vert {\Phi \biggl( { \frac{{\sigma ( y )}}{{{\sigma_{n}} ( y )}}x} \biggr) - \Phi ( x )} \biggr\vert + \frac{{\sqrt{n{h_{n}}} \vert {E{f_{n}} ( y ) - f ( y )} \vert }}{{ \sqrt{2\pi} \sigma ( y )}}. \end{aligned}$$

Here we used the fact that the event $\frac{{\sqrt{n{h_{n}}} }}{{\sigma ( y )}}\vert {E{f_{n}} ( y ) - f ( y )} \vert > a$ does not happen for the selected *a*.

From the inequality $\sup_{y} \vert {\Phi ( {\eta y} ) - \Phi ( y )} \vert \leq\frac{1}{{e\sqrt{2\pi } }} ( {\vert {\eta - 1} \vert + \vert {{\eta^{ - 1}} - 1} \vert } )$, it can be concluded that

4.49 $$ \sup_{x \in\mathbb{R}} \biggl\vert {\Phi \biggl( { \frac {{\sigma ( y )}}{{{\sigma_{n}} ( y )}}x} \biggr) - \Phi ( x )} \biggr\vert = O \bigl( {\bigl\vert {\sigma_{n}^{2} ( y ) - {\sigma^{2}} ( y )} \bigr\vert } \bigr). $$

Under Assumptions A3(ii) and A3(iii), use of the Taylor expansion yields

4.50 $$ \bigl\vert {E{f_{n}} ( y ) - f ( y )} \bigr\vert =O \bigl( h_{n}^{2} \bigr). $$

So from (4.48), (4.49), (4.50), Theorem 2, and Lemma 2, we have

$$\begin{aligned} \sup_{x \in\mathbb{R}} \bigl\vert {\mathrm{P} \bigl( { \sqrt{n{h_{n}}} \bigl[ {{{\hat{f}}_{n}} ( y ) - f ( y )} \bigr] \le x\sigma ( y )} \bigr) - \Phi ( x )} \bigr\vert =& O \bigl( {{a_{n}} + {b_{n}} + h_{n}^{2}} \bigr) \\ =& O \bigl( a'_{n} \bigr). \end{aligned}$$

 □

### Proof of Corollary 1

Using the triangular inequality it can be seen that

4.51 $$\begin{aligned}& \sup_{y \in\mathcal{C}} \bigl\vert {\hat{\sigma}_{n}^{2} ( y ) - {\sigma^{2}} ( y )} \bigr\vert \\& \quad = \sup_{y\in\mathcal{C}} \biggl\vert {\frac{{{\alpha_{n}}{{\hat{f}}_{n}} ( y )}}{{{G_{n}} ( y )}} - \frac{{\alpha f ( y )}}{{G ( y )}}} \biggr\vert \int_{ - 1}^{1} {K ( t )\,dt} \\& \quad \le \sup_{y\in\mathcal{C}} \frac{{G ( y )\vert {{\alpha_{n}}{{\hat{f}}_{n}} ( y ) - \alpha f ( y )} \vert + \alpha f ( y )\vert {{G_{n}} ( y ) - G ( y )} \vert }}{{{G_{n}} ( y )G ( y )}} \int_{ - 1}^{1} {K ( t )\,dt}. \end{aligned}$$

Under Assumptions A3, A5, H1-H4, Theorem 4.1 of [16] we obtain

4.52 $$ \sup_{y\in\mathcal{C}} \bigl\vert {{{\hat{f}}_{n}} ( y ) - f ( y )} \bigr\vert = O \biggl\{ {\max \biggl( { \sqrt{\frac{{\log n}}{{n{h_{n}}}}} ,h_{n}^{2}} \biggr)} \biggr\} \quad \mbox{a.s.} $$

From (4.45) and (4.52) we have

4.53 $$\begin{aligned} \sup_{y\in\mathcal{C}} \bigl\vert {{\alpha_{n}} {{\hat{f}}_{n}} ( y ) - \alpha f ( y )} \bigr\vert =& \sup _{y\in\mathcal{C}} \bigl\vert {{\alpha_{n}} {{\hat{f}}_{n}} ( y ) - {\alpha_{n}}f ( y ) + {\alpha_{n}}f ( y ) - \alpha f ( y )} \bigr\vert \\ \le& \sup_{y\in\mathcal{C}} {\alpha_{n}}\bigl\vert {{{\hat{f}}_{n}} ( y ) - f ( y )} \bigr\vert + \sup_{y\in\mathcal{C}} f ( y )\vert {{\alpha_{n}} - \alpha} \vert \\ =& O \biggl\{ {\max \biggl( {\sqrt{\frac{{\log n}}{{n{h_{n}}}}} ,h_{n}^{2}} \biggr)} \biggr\} + O \biggl( {\sqrt{\frac{{\log\log n}}{n}} } \biggr) \quad \mbox{a.s.} \end{aligned}$$

Using (4.53) and (4.52) in (4.51) proves the corollary. □

### Proof of Theorem 4

Using the triangular inequality we can write

4.54 $$\begin{aligned}& \sup_{x \in\mathbb{R}} \biggl\vert {\mathrm{P} \biggl( { \frac {{\sqrt{n{h_{n}}} ( {{{\hat{f}}_{n}}(y) - f(y)} )}}{{{{\hat{\sigma}}_{n}} ( y )}} \le x} \biggr) - \Phi ( x )} \biggr\vert \\& \quad \le\sup_{x \in\mathbb{R}} \biggl\vert {\mathrm{P} \biggl( { \frac {{\sqrt{n{h_{n}}} ( {{{\hat{f}}_{n}}(y) - E{f_{n}}(y)} )}}{{\sigma (y)}} \le\frac{{{{\hat{\sigma}}_{n}} ( y )}}{{\sigma(y)}}x} \biggr) - \Phi \biggl( { \frac{{{{\hat{\sigma}}_{n}} ( y )}}{{\sigma(y)}}x} \biggr)} \biggr\vert \\& \quad\quad{} + \sup_{x \in\mathbb{R}} \biggl\vert {\Phi \biggl( { \frac{{{{\hat{\sigma}}_{n}} ( y )}}{{\sigma(y)}}x} \biggr) - \Phi ( x )} \biggr\vert . \end{aligned}$$

By Assumptions A1-A3(i), A4 and A5, Theorem 3 results in the following:

4.55 $$ \sup_{x \in\mathbb{R}} \biggl\vert {\mathrm{P} \biggl( { \frac {{\sqrt{n{h_{n}}} ( {{{\hat{f}}_{n}}(y) - E{f_{n}}(y)} )}}{{\sigma (y)}} \le\frac{{{{\hat{\sigma}}_{n}} ( y )}}{{\sigma(y)}}x} \biggr) - \Phi \biggl( { \frac{{{{\hat{\sigma}}_{n}} ( y )}}{{\sigma(y)}}x} \biggr)} \biggr\vert =O\bigl(a'_{n} \bigr), $$

in which $a'_{n}$ is defined in Theorem 3.

Under Assumptions A3, A5, H1-H4, Corollary 1 results in the following:

4.56 $$\begin{aligned} \sup_{x \in\mathbb{R}} \biggl\vert {\Phi \biggl( { \frac {{{{\hat{\sigma}}_{n}} ( y )}}{{\sigma(y)}}x} \biggr) - \Phi ( x )} \biggr\vert =& O \bigl( {\bigl\vert {\hat{\sigma}_{n}^{2} ( y ) - {\sigma^{2}}(y)} \bigr\vert } \bigr) \\ =& O \biggl\{ {\max \biggl( {\sqrt{\frac{{\log n}}{{n{h_{n}}}}} ,h_{n}^{2}} \biggr) + \sqrt{\frac{{\log\log n}}{n}} } \biggr\} \quad \mbox{a.s.} \end{aligned}$$

Substituting (4.55) and (4.56) in (4.54) proves the theorem. □

## Conclusions

In this paper we obtained Berry-Esseen type bounds for the kernel density estimator based on left-truncated and strongly mixing data. Here it is concluded that in RLTM, which is also dealing with weak dependency, we can get asymptotic normality but comparing the results with [11] we see that the rates get much more complicated and also slower.
